# Supplementary material for: Mapping the evolution of fertility support policies in China: A content and instrumental analysis
Source: PLoS One. 2025 Oct 9;20(10):e0332137. doi: 10.1371/journal.pone.0332137 (PMC12510515; doi:10.1371/journal.pone.0332137)
Supplement: S1 Appendix — (ZIP) [file pone.0332137.s001.zip › S1 Appendix. 226 original policy documents/96-民政部、国家发展和改革委员会关于印发《“十四五”民政事业发展规划》的通知(FBM-CLI-4-5015719).doc]

民政部、国家发展和改革委员会关于印发《“十四五”民政事业发展规划》的通知

**发布部门：** [民政部](https://www.pkulaw.com/law/chl?Aggs.IssueDepartment=60208) [国家发展和改革委员会(含原国家发展计划委员会、原国家计划委员会)](https://www.pkulaw.com/law/chl?Aggs.IssueDepartment=60301) [机构沿革](javascript:void(0))

**发文字号：**民发〔2021〕51号

**发布日期：**2021.05.24

**实施日期：**2021.05.24

**时效性：** [现行有效](https://www.pkulaw.com/law/chl?Aggs.TimelinessDic=01)

**效力级别：** [部门规范性文件](https://www.pkulaw.com/law/chl?Aggs.EffectivenessDic=XE0302)

**法规类别：** [计划综合规定](https://www.pkulaw.com/law/chl?Aggs.Category=02401) [营商环境优化](https://dobusiness.pkulaw.com/)

民政部、国家发展和改革委员会关于印发《“十四五”民政事业发展规划》的通知
（民发〔2021〕51号）

各省、自治区、直辖市民政厅（局）、发展改革委，各计划单列市民政局、发展改革委，新疆生产建设兵团民政局、发展改革委：
　　为深入学习贯彻习近平新时代中国特色社会主义思想，全面贯彻党的十九大和十九届二中、三中、四中、五中全会精神，积极推动民政事业更好发挥在社会建设中的兜底性、基础性作用，依据《[中共中央关于制定国民经济和社会发展第十四个五年规划和二〇三五年远景目标的建议](https://www.pkulaw.com/chl/62a4170c41f8f103bdfb.html)》和《[中华人民共和国国民经济和社会发展第十四个五年规划和2035年远景目标纲要](https://www.pkulaw.com/chl/dad03bd96074290cbdfb.html)》，特制定《“十四五”民政事业发展规划》。现印发给你们，请结合实际认真贯彻执行。

民 政 部
　　国家发展和改革委员会
　　2021年5月24日

“十四五”民政事业发展规划
目　录

　　第一章　深入学习贯彻习近平新时代中国特色社会主义思想，开启民政事业现代化建设新征程
　　第一节　站在新的历史起点
　　第二节　肩负新的时代使命
　　第三节　总体思路
　　第四节　发展目标
　　第二章　全力提升基本民生保障水平，在扎实推动共同富裕中作出新贡献
　　第一节　实现巩固拓展脱贫攻坚兜底保障成果同乡村振兴有效衔接
　　第二节　改革完善社会救助制度
　　第三节　提升儿童福利保障水平
　　第四节　完善残疾人福利制度
　　第五节　发挥福利彩票支持保障作用
　　第六节　推动西藏新疆民政事业发展
　　第三章　全面加强和创新基层社会治理，助力国家治理效能实现新提升
　　第一节　夯实基层社会治理基础
　　第二节　深化城乡社区服务体系建设
　　第三节　推动社会组织健康有序发展
　　第四节　完善现代社会工作制度
　　第五节　促进慈善事业健康发展
　　第六节　健全志愿服务体系
　　第七节　加强行政区划和行政区域界线管理
　　第四章　全方位强化基本社会服务，推动民生福祉达到新水平
　　第一节　提升婚姻收养管理服务水平
　　第二节　健全未成年人保护体系
　　第三节　加强困境儿童保障和留守儿童、留守妇女关爱
　　第四节　推进地名管理和公共服务体系建设
　　第五节　提高康复辅助器具助老助残能力
　　第六节　完善殡葬公共服务体系
　　第五章　全要素构建养老服务体系，在实施积极应对人口老龄化国家战略中彰显新作为
　　第一节　加强养老服务保障
　　第二节　优化居家社区机构养老服务网络
　　第三节　壮大养老服务产业
　　第四节　加强养老服务人才队伍建设
　　第五节　提升综合监管水平
　　第六章　强化实施保障，推进民政事业高质量发展
　　第一节　全面推进民政系统党的建设
　　第二节　加强民政法治化标准化建设
　　第三节　提升民政信息化水平
　　第四节　优化民政资源配置
　　第五节　统筹民政领域发展和安全
　　第六节　完善规划实施监测评估机制
　　为明确“十四五”时期民政事业发展主要目标、重点任务和重大举措，依据《[中共中央关于制定国民经济和社会发展第十四个五年规划和二〇三五年远景目标的建议](https://www.pkulaw.com/chl/62a4170c41f8f103bdfb.html)》和《[中华人民共和国国民经济和社会发展第十四个五年规划和2035年远景目标纲要](https://www.pkulaw.com/chl/dad03bd96074290cbdfb.html)》，制定本规划。

　第一章　深入学习贯彻习近平新时代中国特色社会主义思想，开启民政事业现代化建设新征程

　第一节　站在新的历史起点

党的十八大以来特别是“十三五”时期，以习近平同志为核心的党中央高度重视民政工作。习近平总书记多次作出重要指示批示，充分肯定了民政事业发展取得的成绩。党中央、国务院系列会议、重要文件对民政工作进行了全面部署。全国民政系统坚持以习近平新时代中国特色社会主义思想为指导，认真贯彻落实习近平总书记重要指示批示精神和党中央、国务院决策部署，全面践行以人民为中心的发展思想，革弊鼎新、攻坚克难，凝神聚力兜底线、保稳定、促发展，“十三五”规划确定的主要目标任务全面完成，基本民生保障、基层社会治理、基本社会服务等各项工作取得新的重大进展，有力服务了改革发展稳定大局。
　　民政法规制度体系不断健全。《[中华人民共和国慈善法](https://www.pkulaw.com/chl/5df107e39adb063fbdfb.html)》、《[志愿服务条例](https://www.pkulaw.com/chl/49a4eb2d1000a625bdfb.html)》、《[行政区划管理条例](https://www.pkulaw.com/chl/7846b03cf53df019bdfb.html)》等法律法规相继颁布实施，《[中华人民共和国老年人权益保障法](https://www.pkulaw.com/chl/60ff5933d32dc7ccbdfb.html)》、《[中华人民共和国城市居民委员会组织法](https://www.pkulaw.com/chl/74b469aedc9b119fbdfb.html)》、《[中华人民共和国村民委员会组织法](https://www.pkulaw.com/chl/09d5ef876a515fe8bdfb.html)》、《[中华人民共和国未成年人保护法](https://www.pkulaw.com/chl/883cecd7fe38636dbdfb.html)》等适时修改，民政事业健康发展的法治保障得到巩固加强。《关于改革完善社会救助制度的意见》、《[关于加强和完善城乡社区治理的意见](https://www.pkulaw.com/chl/ee5e7482a36e73bcbdfb.html)》、《[关于改革社会组织管理制度促进社会组织健康有序发展的意见](https://www.pkulaw.com/chl/39f2c1ddb0e8b91ebdfb.html)》、《[关于推进养老服务发展的意见](https://www.pkulaw.com/chl/7d8c9213bfaa1e15bdfb.html)》、《[关于加快发展康复辅助器具产业的若干意见](https://www.pkulaw.com/chl/ba90dee173620a3dbdfb.html)》等一系列重要文件制定出台，强化了民政事业的顶层设计。养老服务、农村留守儿童关爱保护和困境儿童保障工作、康复辅助器具产业等部际联席会议制度开创建立，社会救助、社区建设、行政区域界线管理、地名管理工作等部际联席会议制度继续发挥积极作用，民政工作统筹协调机制更加健全。民政工作规范化、标准化水平明显提高。
　　民政工作服务保障国家大局成效明显。坚决扛起脱贫攻坚兜底保障政治责任，创新优化政策供给，2017年底全国所有县（市、区、旗）农村低保标准均已达到或动态超过国家扶贫标准，共有1936万脱贫人口纳入兜底保障范围，脱贫攻坚“兜底一批”任务圆满完成。民政系统援藏援疆工作为西藏、新疆社会稳定和长治久安夯实民生基础。对中西部地区、特殊类型地区等倾斜支持力度持续加大，在深入实施区域重大战略、区域协调发展战略等国家战略中积极贡献民政方案。社会组织有序发展，社区服务体系更加完善，行政区划资源效应越发凸显，民政工作服务经济社会发展愈加有效。全系统抗击新冠肺炎疫情取得重大成果，有效保障民政服务对象生命安全和身体健康，引导社会力量踊跃捐款捐物，动员社会工作者、志愿者主动投身疫情防控，400多万名社区工作者坚守65万个城乡社区，筑牢了基层群防群治的坚固防线。
　　基本民生保障兜底工作提质增效。与经济社会发展水平相适应的民政兜底保障体系基本建成，各类困难群众基本生活得到有效保障和改善。到“十三五”末，城乡居民最低生活保障平均标准分别达到678元/人·月和5962元/人·年，比“十二五”末分别提高50.3%和87.6%。孤儿基本生活保障水平持续提升，25.4万名事实无人抚养儿童全面纳入国家保障范围。困难残疾人生活补贴和重度残疾人护理补贴制度覆盖全部县（市、区、旗），发放人数分别达到1212.6万人和1473.8万人，补贴标准动态调整机制初步建立。特困人员救助供养制度实现城乡统筹，临时救助制度有效实施。流浪乞讨人员人身安全和基本生活得到有效保障，寻亲水平明显提升，安置渠道更加通畅。
　　基层社会治理新格局加快形成。基层民主制度化、规范化、程序化建设持续加强，基层群众性自治组织特别法人制度建立并不断完善，城乡社区协商内容逐步充实、形式更加丰富、主体日益广泛，与其他协商形式衔接更加紧密。城乡社区综合服务设施建设稳步推进，社区社会组织作用积极发挥，社区服务能力不断提升。全国社会组织数量达到89.4万个，吸纳社会各类人员就业超过1000万人，均比“十二五”末增长30%以上。全国社会工作专业人才总量达到157.3万人，其中持证社会工作者66万人；注册志愿者人数超过1.92亿人。慈善信托等新制度为社会力量参与慈善事业开辟新途径，社会捐助实现快速发展。城乡社区、社会组织、社会工作“三社联动”效果显著，党组织领导的自治、法治、德治相结合的基层社会治理新格局加快形成。
　　基本社会服务和专项行政管理水平持续提升。养老服务发展步伐加快，全国养老机构和设施总数达到31.9万个，各类养老床位数达到823.8万张。婚姻登记收费全面取消，《[中华人民共和国民法典](https://www.pkulaw.com/chl/aa00daaeb5a4fe4ebdfb.html)》增设“离婚冷静期”制度，婚姻家庭心理疏导、危机干预等辅导服务机制普遍建立。农村留守儿童、妇女和老年人关爱服务体系逐步完善，未成年人保护工作取得突破性进展。惠民殡葬制度建设持续推进，殡葬设施和服务明显改善。行政区划和地名管理进一步优化，依法管界护界工作深入开展，第二次全国地名普查圆满完成。
　　党的建设持续从严加强。严格落实全面从严治党主体责任，持续完善党建工作制度。认真开展“两学一做”学习教育、“不忘初心、牢记使命”主题教育。推进党建工作同业务工作深度融合，推动全面从严治党向纵深发展。坚定不移开展党风廉政建设和反腐败斗争，深入查处福利彩票系列腐败案件，规范福利彩票发行销售管理，中央巡视整改任务全面完成。聚焦农村低保、疫情防控、殡葬等重点领域开展专项监督，持之以恒推动脱贫攻坚专项巡视、“回头看”整改，全面完成整改任务。风清气正的良好政治生态和干事创业环境巩固提升，有力保障了民政事业健康发展和民政干部健康成长。
　　同时也要看到，与全面建设社会主义现代化国家的更高要求相比，与党中央、国务院对民政工作的决策部署相比，与人民日益增长的美好生活需要相比，社会救助、养老服务、儿童福利、未成年人保护、残疾康复、精神卫生、殡葬服务等领域的基本民生保障和基本社会服务短板亟待补齐，基层社会治理中社会力量参与不畅、社区服务能力不足等弱项仍然存在，城乡区域间民政服务能力不平衡的问题依然突出，支撑民政事业持续健康发展的标准化、信息化建设亟待加强，推动新时代民政事业高质量发展任重道远。

　第二节　肩负新的时代使命

进入新发展阶段，必须完整、准确、全面贯彻新发展理念，在构建新发展格局中抢抓机遇，迎接挑战，扛起责任，奋勇前进，为全面建设社会主义现代化国家贡献力量。
　　习近平总书记重要指示为新时代新阶段民政事业发展指明了新航向。习近平总书记明确指出：“民政工作关系民生、连着民心，是社会建设的兜底性、基础性工作”、“各级民政部门要加强党的建设，坚持改革创新，聚焦脱贫攻坚，聚焦特殊群体，聚焦群众关切，更好履行基本民生保障、基层社会治理、基本社会服务等职责，为全面建成小康社会、全面建设社会主义现代化国家作出新的贡献”。习近平总书记的重要指示深刻指出了新时代民政工作的地位作用、基本职责、根本保障、发展动力和使命任务，科学回答了新时代新阶段如何定位民政工作、如何做好民政工作等重大问题，为民政事业改革发展指明了前进方向，提供了根本遵循。
　　党中央、国务院重要决策部署为新时代新阶段民政事业发展描绘了新蓝图。党的十九大和十九届二中、三中、四中、五中全会对民政工作作出战略部署，党中央、国务院在一系列重要会议和文件中对民政工作作出系统安排，对民政事业发展提出了新的更高要求，为不断健全完善基本民生保障制度体系，加快形成党组织领导的自治、法治、德治相结合的城乡基层社会治理新格局，推动养老、殡葬等基本社会服务更加均等可及明确了时间表、任务书和路线图，为民政工作更好发挥社会建设兜底性、基础性作用作出了顶层设计。
　　我国经济和科技实力为新时代新阶段民政事业发展提供了新支撑。“十三五”时期，我国经济总量跃上百万亿元新的大台阶，人均国内生产总值超过1万美元，5575万农村贫困人口全部脱贫，城镇化率超过60%。“十四五”时期，我国有望迈进高收入国家行列，民政事业发展的经济基础将进一步巩固。人工智能、生命健康等前沿科技，新一代信息技术、大数据等创新应用，正在催生民政领域一系列新技术、新产品、新业态、新模式，为基本民生保障对象主动发现和精准识别、基层社会治理数字化和智慧化、基本社会服务供给和需求适配，提供了新的支撑和有效路径。
　　新的历史方位对新时代新阶段民政事业发展提出了新要求。进入新发展阶段，在贯彻落实巩固脱贫攻坚成果同乡村振兴有效衔接、积极应对人口老龄化、区域协调发展、以人为核心的新型城镇化、基本公共服务均等化等党和国家重大战略任务和重大决策部署中，民政事业面临现代化建设新征程中的新任务。贯彻新发展理念，供给侧结构性改革的任务更加迫切，更加需要强基础、转方式、拓领域，采取有效举措切实缩小不平衡差距，强化资源集约利用，加大对外合作交流，在增进民生福祉推进共同富裕中展现新作为。构建新发展格局，高品质多样化的民生需求对内需体系的支撑作用更加显著，保障和改善基本民生、推进基本社会服务均等普惠、加强和创新社会治理有利于培育形成新的增长点，促进民政事业同经济发展有效衔接、良性循环。

　第三节　总体思路

高举中国特色社会主义伟大旗帜，深入贯彻党的十九大和十九届二中、三中、四中、五中全会精神，坚持以马克思列宁主义、毛泽东思想、邓小平理论、“三个代表”重要思想、科学发展观、习近平新时代中国特色社会主义思想为指导，全面贯彻党的基本理论、基本路线、基本方略，深入贯彻落实习近平总书记关于民政工作重要指示精神，科学把握新发展阶段，坚决贯彻新发展理念，服务构建新发展格局，以推动民政事业高质量发展为主题，以充分发挥民政工作在社会建设中的兜底性、基础性作用为主线，以改革创新为根本动力，以满足人民日益增长的美好生活需要为根本目标，更好履行基本民生保障、基层社会治理、基本社会服务等职责，突出政治性、群众性、时代性、协同性，统筹发展和安全，为全面建设社会主义现代化国家作出新的贡献。
　　“十四五”时期，民政工作必须坚持以下原则：
　　--坚持党的全面领导。贯彻党把方向、谋大局、定政策、促改革的要求，深入学习习近平新时代中国特色社会主义思想，始终把习近平总书记重要指示精神作为民政工作的根本遵循，贯彻落实新时代党的组织路线，坚持严的主基调，确保民政事业始终沿着正确政治方向前进。
　　--坚持以人民为中心。聚焦巩固拓展脱贫攻坚兜底保障成果，聚焦特殊群体，聚焦群众关切，科学界定基本民生保障范围，稳步提升基本社会服务水平，尽力而为、量力而行，将党和政府的惠民政策精准送到群众身边，为实现“全体人民共同富裕取得更为明显的实质性进展”作出新贡献。
　　--坚持共建共治共享。充分调动广大社会组织、社会工作者、志愿者和慈善组织等社会力量，更好发挥政府作用，统筹利用各类资源，加大向基层放权力度，向基层队伍赋能、向参与主体赋能、向服务对象赋能，促进广泛参与、各负其责、互为补充、同频共振。
　　--坚持深化改革创新。用改革的办法和开放的视野补短板、强弱项、破壁垒，聚焦重点领域和关键环节，全面深化社会救助、基层治理、残疾人福利、儿童福利、养老服务、殡葬等改革，鼓励基层开拓创新，探索开展原创性、差异化改革，不断为民政事业发展探索新方式、注入新动能。
　　--坚持系统观念。以全局站位和长远眼光推动民政工作融入区域协调发展、创新驱动发展、乡村振兴、新型城镇化、可持续发展等国家重大战略，坚持民政事业城乡统筹、区域协调、整体推进，加强跨业务、全系统、多部门协同，更加注重防范化解民政领域重大风险，推动民政事业行稳致远。

　第四节　发展目标

到2035年民政事业发展的远景目标：对标国家基本实现社会主义现代化远景目标，展望2035年，与社会主义现代化国家相匹配的民政法规制度体系、技术支撑体系、人才队伍体系等全面形成，以人民为中心、共建共治共享、精准高效协同、兼顾韧性和温度的民政事业高质量发展新局面全面形成；基本民生保障体系更加完善，精准覆盖全部目标群体，在推进全体人民共同富裕中提供坚实兜底保障；基层社会治理能力基本实现现代化，在国家治理体系和治理能力现代化建设和更高水平平安中国建设中发挥重要基础性作用；基本社会服务实现更高层次、更高水平发展，成为国家基本公共服务均等化进程的重要支撑，在满足人民美好生活需要中贡献重要力量。
　　到2025年民政事业发展主要目标：民政系统党的建设全面加强，制度体系进一步健全完善，基本民生保障、基层社会治理、基本社会服务水平显著提高，城乡区域发展差距明显缩小，民政事业现代化建设取得明显进展。
　　--基本民生保障更加有力。实现巩固拓展脱贫攻坚兜底保障成果同乡村振兴有效衔接。社会救助制度进一步完善，分层分类的社会救助体系建立健全。老年人、残疾人、儿童等社会福利适度普惠迈出新步伐。福利彩票支持基本民生保障的能力和水平进一步提升。
　　--基层社会治理更加有序。基层群众自治制度不断完善，城乡社区服务体系更加健全。社会组织、社会工作者、志愿者联动机制和参与社会治理的途径进一步畅通和规范。慈善事业的第三次分配作用更加凸显。行政区划设置与推进区域协调发展、新型城镇化进程相适应。
　　--基本社会服务更加有效。基本养老服务制度全面建立，居家社区机构相协调、医养康养相结合的养老服务体系更加完善，未成年人保护工作机制更加系统完备，地名、婚姻、殡葬等服务管理更加科学有效，普惠均等和智能精准程度同步提高。

| “十四五”时期民政事业主要发展指标 | | | | |
| --- | --- | --- | --- | --- |
| 序号 | 指标 | 单位 | 2025年目标值 | 属性 |
| 1 | 城乡低保标准年度增速 | % | 不低于居民上年度人均消费支出增速 | 预期性 |
| 2 | 农村低保标准占城市低保标准比例 | % | 75 | 预期性 |
| 3 | 困难残疾人生活补贴覆盖率 | % | 100 | 约束性 |
| 4 | 重度残疾人护理补贴覆盖率 | % | 100 | 约束性 |
| 5 | 福利彩票销售网点数 | 万个 | 18 | 预期性 |
| 6 | 每百户居民拥有的城乡社区综合服务设施面积 | 平方米 | ＞30 | 预期性 |
| 7 | 社会组织专职工作人员数量 | 万人 | 1250 | 预期性 |
| 8 | 社会工作专业人才总量 | 万人 | 200 | 预期性 |
| 9 | 志愿服务站点在社区综合服务设施中的覆盖率 | % | 80 | 预期性 |
| 10 | 婚姻登记“跨省通办”实施率 | % | 100 | 预期性 |
| 11 | 乡镇（街道）未成年人保护工作站覆盖率 | % | 50 | 预期性 |
| 12 | 查明身份信息流浪乞讨受助人员接送返回率 | % | 100 | 约束性 |
| 13 | 县级公益性安葬（放）设施覆盖率 | % | 100 | 约束性 |
| 14 | 养老机构护理型床位占比 | % | ≥55 | 约束性 |
| 15 | 乡镇（街道）范围具备综合功能的养老服务机构覆盖率 | % | 60 | 预期性 |

　第二章　全力提升基本民生保障水平，在扎实推动共同富裕中作出新贡献

坚持以人民为中心，尽力而为、量力而行，推动巩固拓展脱贫攻坚兜底保障成果同乡村振兴有效衔接，健全分层分类的社会救助体系，稳步提升残疾人、孤儿等社会福利水平，不断增强人民群众获得感、幸福感、安全感。

　第一节　实现巩固拓展脱贫攻坚兜底保障成果同乡村振兴有效衔接

巩固拓展脱贫攻坚兜底保障成果。建设低收入人口动态监测信息平台，加强对低保对象、特困人员、低保边缘家庭等易返贫致贫人口，以及因病因灾因意外事故等刚性支出较大或收入大幅缩减导致基本生活出现严重困难人口等农村低收入人口的动态监测，健全常态化救助帮扶机制。保持农村社会救助政策持续稳定，完善农村社会救助制度，调整优化对原建档立卡贫困户的低保“单人户”政策，将未纳入低保或特困供养范围的低保边缘家庭中的重病、重残人员，参照“单人户”纳入低保。鼓励有劳动能力的农村低保对象参与就业，在计算家庭收入时扣减必要的就业成本。完善农村特困人员救助供养制度，合理确定救助供养水平，提高服务质量。加强社会救助资源统筹，根据对象类型、困难程度等，及时有针对性地给予困难群众医疗、教育、住房、就业等专项救助，做到精准识别、应救尽救。对基本生活陷入暂时困难的群众加强临时救助，做到凡困必帮、有难必救。强化县乡两级公办养老机构对失能、部分失能特困老年人的兜底保障。切实保障好农村地区孤儿、事实无人抚养儿童及其他困境儿童等基本生活。严格落实“摘帽不摘责任、摘帽不摘政策、摘帽不摘帮扶、摘帽不摘监管”要求，持续开展民政部门定点帮扶。加强对乡村振兴重点帮扶县的支持。
　　提升农村地区民政公共服务水平。推进社会救助制度城乡统筹发展，加大农村社会救助投入，逐步缩小城乡差距，推进城乡社会救助服务均等化。完善帮扶残疾人、孤儿等社会福利制度，健全农村地区困境儿童和留守老年人、留守儿童、留守妇女关爱服务体系。统筹推进农村民政服务设施和村级综合服务设施建设，促进公共服务资源向农村覆盖，向中西部地区、脱贫地区、民族地区、特殊类型地区等倾斜，推动农村公共服务水平持续提升。加强农村基层社会治理队伍建设。鼓励引导社会组织、社会工作者和志愿服务组织积极向乡村提供民政公共服务，共同推进乡村振兴。

　第二节　改革完善社会救助制度

健全社会救助制度体系。积极推动社会救助法制定出台。完善低保制度，制定低保审核确认办法，规范低保对象认定程序，提高科学性和精准度。加快推进城乡低保统筹发展，有条件的地区积极推行持有居住证人员在居住地申请低保。加强对低保边缘家庭和支出型困难家庭的救助工作，分层分类实施社会救助。完善特困人员认定办法，将特困人员救助供养覆盖的未成年人年龄从16周岁延长至18周岁。做好残疾儿童康复救助、重度残疾人护理补贴、孤儿保障、事实无人抚养儿童保障等制度与社会救助政策有效衔接。强化急难社会救助功能，对遭遇突发事件、意外伤害、重大疾病、受传染病疫情等突发公共卫生事件影响或其他特殊原因导致基本生活暂时陷入困境，靠自身和家庭无力解决，其他社会救助制度暂时无法覆盖或救助之后生活仍有困难的家庭或个人，通过临时救助或生活无着的流浪乞讨人员救助给予应急性、过渡性生活保障。探索由急难发生地实施临时救助。畅通急难情况及时报告、主动发现渠道，健全快速响应、个案会商“救急难”工作机制。建立容错纠错机制，对秉持公心、履职尽责但因客观原因出现失误偏差且能够及时纠正的经办人员依法依规免于问责。
　　完善基本生活救助标准动态调整机制。完善低保标准制定办法，建立与居民人均消费支出或人均可支配收入水平挂钩的动态调整机制。以当地低保标准为基础，上浮一定比例确定特困人员基本生活标准，原则上不低于1.3倍；根据生活自理能力和服务需求分档制定照料护理标准。推动以省（自治区、直辖市）为单位研究制定相对统一的区域救助标准，逐步缩小城乡区域差距。完善社会救助和保障标准与物价上涨挂钩联动机制。
　　强化社会救助家庭经济状况核对机制建设。完善低保家庭收入财产认定方法，制定与经济社会发展水平相适应的低保家庭财产限定标准或条件。推进民政与教育、公安、人力资源社会保障、自然资源、住房城乡建设、卫生健康、市场监管、医疗保障、金融、乡村振兴、残联等部门和机构信息互联互通、数据共享共用，依法明确和落实相关部门、机构提供社会救助家庭收入及财产信息的责任。完善核对内容，优化核对系统，逐步推进全国联网的统一核对工作。强化信息技术支撑，加强核对系统与社会救助信息系统的协同，建立无缝衔接、精准高效的社会救助对象认定机制。
　　扩大社会救助服务供给。积极发展服务类社会救助，拓展“物质＋服务”的救助方式，为有需要的救助对象提供心理疏导、社会融入、资源链接等服务，增强困难群众内生动力和自我发展能力。探索通过政府购买服务等方式为社会救助家庭中生活不能自理的老年人、未成年人、残疾人等提供必要的访视、照料服务。加强社会工作服务，帮助救助对象构建家庭和社会支持网络。
　　深化社会救助“放管服”改革。全面推行“一门受理、协同办理”，由乡镇（街道）经办机构统一受理社会救助申请，并按照职责分工及时办理或转请县级相关职能部门办理。优化审核确认程序，有条件的地方可按程序将低保、特困等社会救助审核确认权限下放至乡镇（街道），取消可以通过国家或地方政务服务平台查询的相关证明材料。加快服务管理转型升级，推进现代信息技术在社会救助领域的运用，完善社会救助资源库，推动社会救助服务向移动端延伸。
　　保障流浪乞讨人员人身安全和基本生活。推动建立健全政府负责同志牵头的救助管理工作领导协调机制，明确部门职责，强化协同联动。充分发挥平安建设（综治工作）考核评价体系作用，提升救助管理水平。完善救助管理政策体系和标准体系，制定落户安置、源头治理等配套政策，强化站内照料职责，加强托养机构监管，切实保障受助人员人身安全和基本生活。健全流浪乞讨人员转介处置机制，减少不文明流浪乞讨现象。推进救助寻亲服务，充分利用智能化手段，发动社会力量，帮助滞留人员回归家庭和社会。推动符合条件的长期滞留人员落户安置并享受相应社会保障。推进源头治理，建立易流浪走失人员信息库，减少反复流浪乞讨现象。升级完善救助管理信息系统，改造全国救助寻亲网。

专栏1 基层社会救助服务能力提升工程

01 提升基层社会救助经办服务能力

整合社会救助资源，强化乡镇（街道）社会救助责任，提升保障条件。村级设立

社会救助协理员，困难群众较多的村（社区）建立社会救助服务站（点），将走

访、发现需要救助的困难群众列为村（社区）重要工作内容。加强政府购买社会

救助服务，为有需要的困难群众提供主动发现和协助申请、资源链接、政策信息、

心理调适、转办转介、社会融入等相关服务。优化社会救助程序，取消可以通过

国家或地方政务服务平台查询的相关证明材料。开通社会救助服务热线，逐步实

现全国联通。

02 完善流浪乞讨人员救助管理设施体系

推动尚无救助管理设施或设施设备落后的地级市，以及人口密集、交通便利、省

市交界、救助量大的县（市、区、旗），新建或改扩建救助管理设施，配齐相关

设施设备。充分利用现有社会福利设施建设流浪乞讨人员救助管理设施或救助管

理站，实现救助管理服务网络覆盖全部县（市、区、旗）。

　第三节　提升儿童福利保障水平

完善帮扶孤儿和事实无人抚养儿童福利制度。规范落实事实无人抚养儿童认定标准和程序。推动建立孤儿和事实无人抚养儿童基本生活保障标准动态调整机制，推进落实孤儿和事实无人抚养儿童保障标准自然增长机制，确保保障标准与经济社会发展水平相匹配、与相关社会福利标准相衔接。持续提高孤儿和事实无人抚养儿童在医疗、教育、康复、就业等方面的保障水平，拓展保障范围。孤儿和事实无人抚养儿童成年后，对符合城镇住房保障条件的，及时纳入保障范围。严格规范家庭寄养。优化完善社会散居孤儿、家庭寄养儿童、事实无人抚养儿童家庭走访、家庭培训和监护评估、监护保护制度。探索对符合条件的事实无人抚养儿童开展助医助学工作。
　　推进儿童福利机构优化提质。加快推进儿童福利机构养育、医疗、教育、康复和社会工作一体化发展，拓展服务范围，提升服务质量。地级以上儿童福利机构应当集中养育本辖区由民政部门长期监护的儿童，省级民政部门可统筹确定养育儿童较多、综合条件较好的部分县级儿童福利机构集中养育由民政部门长期监护的儿童。推动每个省（自治区、直辖市）培育2家以上儿童福利机构高质量发展示范单位。推进建设辐射多省份的区域性儿童福利机构。支持儿童福利机构建造家庭式居所，推广家庭式养育模式。推动儿童福利机构资源共享、合作交流，加强儿童福利机构与专业康复机构、医疗机构、特教机构合作，提高机构服务水平和儿童生活质量。鼓励有条件的儿童福利机构向社会残疾儿童拓展服务。会同有关部门，保障机构内适合就学的适龄残疾儿童全部接受教育并纳入学籍管理。推动儿童福利机构均达到定点康复机构标准，支持符合条件的纳入残疾儿童康复救助定点机构目录。
　　加强儿童福利工作标准化建设。推动成立全国儿童福利工作标准化技术委员会，组织制定一批适应儿童福利工作的标准，推动地方标准、行业标准上升为国家标准，建立全国统一的服务质量标准和评价体系。

专栏2 儿童福利服务提质工程

01 加强儿童福利机构建设

重点支持有集中养育需求、设施设备落后的地区新建或改扩建儿童福利机构，推

动儿童福利机构优化提质，完善儿童养育、残疾儿童医疗、特教、康复及开展家

庭式养育等所需的设施设备，推动县级儿童福利机构转型为未成年人救助保护机

构。

02 提升儿童福利服务能力

开展孤残儿童护理员、儿童福利院院长入职培训和定期培训，建立健全孤残儿童

护理员职业技能等级认定制度。支持有条件的地区在儿童福利服务人才培训等方 面给予政策倾斜。培育孵化儿童福利类社会组织，推动建立中国儿童福利联合会。推动儿童福利服务纳入政府购买服务目录，支持社会工作服务机构开展儿童福利服务。

　第四节　完善残疾人福利制度

健全残疾人“两项补贴”动态调整机制。各地根据经济社会发展情况及残疾人实际需求，建立困难残疾人生活补贴和重度残疾人护理补贴标准动态调整机制。逐步推进困难残疾人生活补贴覆盖至低保边缘家庭和其他困难残疾人，重度残疾人护理补贴覆盖至三、四级智力和精神残疾人。积极推进残疾人“两项补贴”资格认定申请跨省通办，为残疾人提供更加贴心暖心的服务。加强残疾人“两项补贴”制度与长期护理保险、老年人福利、社会救助等制度相衔接。鼓励有条件的地区探索新的补贴内容和形式。
　　推动精神障碍康复服务向社区延伸。发挥精神卫生专业机构技术指导作用，推动社区精神障碍康复机构与精神卫生福利、残疾人康复、残疾人托养、社区卫生服务等机构服务对接、场地共用、资源共享，培育一批社区精神障碍康复标杆机构，发挥示范引领作用，形成一套“经验包”在全国推广，逐步将社区精神障碍康复机构打造成康复服务网络中的重要节点。支持社会力量参与社区精神障碍康复机构建设，鼓励有条件的地区在服务设施、运行补贴、职称待遇等方面给予扶持，将居家康复、照护技能培训纳入精神障碍社区康复基本服务范围，为家庭提供照护资讯、政策咨询、情感支持等专业服务，巩固和增强家庭照护功能，促进家庭成为精神障碍社区康复服务体系的重要力量和资源。

专栏3 精神卫生福利设施建设工程

在精神卫生服务能力不足的地市建设100个左右精神卫生福利设施，支持需求大

的县（市、区、旗）建设精神卫生福利设施，为特殊困难精神障碍患者提供集中

养护、康复等服务。积极引导和鼓励社会力量参与精神卫生福利设施建设和运营。

　第五节　发挥福利彩票支持保障作用

保障发行销售安全健康运行。健全行业标准体系，完善管理制度，强化制度执行。健全行业激励约束机制、市场调控机制和监督管理机制，不断强化福利彩票发行销售整体性、协同性。建立健全风险预警、防控和安全管理机制，提升风险防控和处置能力。强化技术能力建设，稳步推进发行销售技术系统统一，建立大数据分析和决策支持系统。深化福利彩票社会责任建设，宣传普及彩票知识，防范非理性购彩，保障参与者合法权益。
　　推动发行销售创新发展。坚持公益性、提高娱乐性、增强文化性，加强游戏研发储备，优化产品结构。顺应人民群众文化娱乐消费新趋势，逐步建立规范化精细化的渠道管理体系，推动传统销售网点转型发展，加强渠道创新，提高购彩便利性，培育购彩新习惯。创新营销品牌，加强营销宣传统筹整合力度，开展跨机构、跨票种、跨区域营销活动。积极探索符合公益事业发展规律和市场发展规律的发行销售运营模式，提高运营效率。加强游戏研发、信息技术、营销宣传、市场管理、理论研究等专业人才培养。
　　加强彩票公益金使用管理。完善各级民政部门彩票公益金使用管理全链条制度体系。突出科学性、精准性、时效性，优化资金分配，加大彩票公益金对乡村振兴重点帮扶县的倾斜支持。加强对彩票公益金资助项目的分类指导，切实践行福利彩票“扶老、助残、救孤、济困”的发行宗旨。实施彩票公益金项目绩效管理，强化审计监督和信息公开，确保资金使用管理规范高效。加大公益宣传力度，创新宣传手段，打造一批公益品牌，展现福利彩票为民、责任、阳光形象，促进彩票公益金使用和发行销售良性循环。

专栏4 福利彩票健康发展工程

01 建设全国统一的发行销售技术系统

积极推动纸质票和电脑票发行销售系统建设，逐步实现分票种全国纵向系统统

一。加强技术标准规范体系建设，加快形成统一渠道管理、统一客户管理、统

一支付管理、统一终端安全管理等技术支撑能力，促进跨票种横向业务融合。

02 推进销售渠道规范化精细化管理

加强渠道规范化建设，制定实施《中国福利彩票销售场所通用要求》。推动传

统销售网点转型发展，优化布局，加强服务，提升形象。拓展新渠道，提高购

彩便利性。加强渠道精细化管理，搭建分类分级管理体系，强化培训和服务，

增强可持续发展能力。

03 提升公益品牌形象

适应融媒体信息传播模式，建立健全品牌建设和营销宣传工作机制，整合资源

开展整体营销。依托销售场所，建设一批集公益宣传、便民服务、彩票销售功

能于一体的“福彩公益驿站”。加大彩票公益金使用宣传力度，强化信息公开，

推动标识设置全覆盖。

　第六节　推动西藏新疆民政事业发展

坚决贯彻落实新时代党的治藏方略和治疆方略。深入贯彻落实中央第七次西藏工作座谈会、第三次中央新疆工作座谈会战略部署，加大对西藏自治区、涉藏工作重点省及新疆维吾尔自治区、新疆生产建设兵团民政事业发展的指导和支持，稳步提高基本民生保障水平，创新开展基层社会治理，持续加大基本社会服务供给，指导优化行政区划设置，助推巩固拓展脱贫攻坚成果同乡村振兴有效衔接，缩小发展差距，推进共同富裕。
　　全面加强民政对口支援。健全民政系统对口支援工作机制。加强支援地与受援地沟通衔接，在政策制定、项目建设、资金支持、干部交流、人才培训等方面创新方式、加大倾斜，推动民政援助项目纳入各级政府对口支援规划，全力推进受援地提升民政工作水平。
　　动员引导社会力量参与对口支援。组织开展全国性社会组织、慈善组织援藏援疆活动。支持西藏自治区、涉藏工作重点省及新疆维吾尔自治区、新疆生产建设兵团社会组织和社会工作发展。强化宣传引导，动员各地社会组织、社会工作服务机构对接需求，发挥优势，广泛开展救助、教育、文化、卫生等慈善公益和志愿服务活动。

　第三章　全面加强和创新基层社会治理，助力国家治理效能实现新提升

夯实基层社会治理基础，强化城乡社区治理和服务体系建设，更好发挥社会组织在社会治理中的作用，畅通社会工作者和志愿者参与社会治理的途径，发展慈善事业，优化行政区划设置，推动建设人人有责、人人尽责、人人享有的基层社会治理共同体。

　第一节　夯实基层社会治理基础

完善党领导的基层群众自治制度。发挥基层群众性自治组织作用，积极推行村（社区）党组织书记“一肩挑”和村（社区）“两委”成员交叉任职。加强基层群众性自治组织规范化建设，建立城乡基层群众自治领域扫黑除恶常态化工作机制。推进减轻村级组织负担。完善基层群众性自治组织出具证明工作的规范化制度。推动社区居民委员会设立环境和物业管理委员会，逐步提高村民委员会成员中女性比例。加强村（居）务监督委员会建设，推进村（居）务公开。
　　拓宽群众参与基层治理的制度化渠道。健全城乡社区协商制度，探索建立乡镇（街道）协商与城乡社区协商的联动机制。推动城乡社区全部制定居民自治章程、村民自治章程和居民公约、村规民约，并在社区治理中有效发挥作用。开展村级议事协商工作，配合开展民主法治示范村（社区）创建、农村人居环境改造等工作，调动群众广泛参与爱国卫生运动，不断推进基层治理改革创新。
　　深化乡镇（街道）服务能力建设和管理创新。强化乡镇（街道）对辖区内公共服务的监督管理，推广“街乡吹哨、部门报到、接诉即办”等基层管理经验，建立乡镇（街道）与县（市、区、旗）有关职能部门之间高效协调机制。积极推行乡镇（街道）政务服务“好差评”机制。深化乡镇（街道）服务管理体制改革，提高“放管服”承接能力，实行“一站式”服务和“一窗式”办理，推动乡镇（街道）一站式服务中心或便民服务、一体化在线政务服务平台实现全覆盖。

　第二节　深化城乡社区服务体系建设

扩大城乡社区服务有效供给。推动社区服务体系建设纳入国民经济和社会发展规划、国土空间规划和新型城镇化建设工程。推动资源、管理、服务向社区下沉，加快建设现代社区。推进城市社区生活性服务综合体建设，深化社区便民惠民服务。开展“新时代新社区新生活”服务质量提升行动。创新城乡社区公共服务提供方式，全面推进政府购买社区服务机制建设，普遍建立政府购买社区服务清单，培育扶持一批具有较强专业性和信誉度的社区服务机构。强化政府引导，支持社会组织、社会工作专业人才、志愿服务组织提供专业化、个性化城乡社区服务。
　　缩小城乡社区服务差距。坚持城乡融合发展，推动城市公共服务资源和慈善、社会工作资源向农村，特别是易地扶贫搬迁集中安置社区及生态移民搬迁安置社区等辐射延伸，推进城乡社区基本公共服务均等化。促进乡村治理与公共服务联动，构建乡镇政府、村民委员会、村集体经济组织和社会组织、村民群众等多主体共同参与的服务格局。
　　加强城乡社区工作者队伍建设。建立社区工作者职业发展体系，建立健全与岗位特点、工作年限、教育程度、专业水平相匹配的社区工作者岗位等级序列，建立社区工作者薪酬体系和待遇正常增长机制，健全完善参加社会保险和缴存住房公积金制度。建立健全社区工作者分级培训制度，加大对中西部地区倾斜，支持社区工作者参加社会工作职业资格考试和学历教育等，推动对获得社会工作职业资格的给予职业津贴。实施大学生社工计划。引导高校毕业生到社区就业创业。推动实现每万名城镇常住人口拥有社区工作者18人。

　第三节　推动社会组织健康有序发展

完善党领导社会组织制度。推动建立各级社会组织工作协调机制，完善社会组织党建工作体制。落实党建工作与登记、年检（年报）、评估“三同步”制度，按照应建尽建原则，持续推进社会组织党的组织和党的工作从“有形覆盖”向“有效覆盖”转化，全面提高社会组织党组织建设质量。培养社会组织专职党务工作者队伍，鼓励社会组织负责人和党组织书记依法依规交叉任职。推行党组织和管理层共同学习、共议重大事项等做法，发挥党组织在社会组织增强职工群众政治认同、依法执业诚信从业中的引导和监督作用。
　　深化社会组织领域改革。巩固深化行业协会商会与行政机关脱钩改革成果，深化行业协会商会改革。依法推进直接登记改革，制定社会组织直接登记办法，强化发起人和负责人资格审查，加强名称审核、业务范围审定。厘清各部门对直接登记社会组织和已脱钩行业协会商会的管理服务职责，构建登记管理机关、党建工作机构、行业管理部门、相关职能部门各司其职、相互配合、依法监管的新型管理体制。加快社会组织法治建设，做好出台《社会组织登记管理条例》有关工作，制定配套政策，推动各行业领域在立法中明确社会组织的权利和义务，提高社会组织工作法治化水平。完善落实社会组织扶持政策，加强基层社会组织管理服务能力建设。
　　提升社会组织服务能力。健全社会组织法人治理结构，完善落实民主选举、民主决策、民主管理、民主监督机制。引导社会组织建立健全财务、人事、资产、活动、分支机构等内部管理制度，提高社会组织依法依章程开展活动的意识和能力，使社会组织成为独立承担法律责任、权责明确、运转协调、制衡有效的法人主体。拓宽扶持政策覆盖面，鼓励有条件地区打造社会组织孵化基地。加大社区社会组织培育扶持力度，动员引导社会组织有序参与社会治理、环境治理，积极提供公共服务，承担社会责任。按照国家规定，推动将社会组织纳入有关部门表彰奖励范围。
　　健全社会组织综合监管体系。落实财务、会计、票据、税务、资产管理政策，完善社会组织非营利监管制度，规范社会组织涉企收费、对外投资、关联交易、募捐捐赠等行为。整治社会组织依托公权力强制入会、开展评比达标表彰活动、党政领导干部在社会组织兼职等行为。会同行业管理部门制定不同行业、领域、层级的社会组织分类管理办法，实现管理对象、管理措施、管理效果精细化。推进社会组织信息公开，鼓励社会监督，强化社会组织信用监管。推动“互联网＋社会组织”制度建设，完成国家社会组织法人库建设。完善清算注销制度，加快清理“僵尸”社会组织。加大执法力度，建立简易处罚程序，完善快速反应机制和综合执法机制，常态化开展非法社会组织打击工作，铲除非法社会组织滋生土壤，防范化解社会组织领域重大风险。

　第四节　完善现代社会工作制度

构建社会工作服务体系。健全党委领导、政府负责、群团助推、社会协同、公众参与的社会工作推进机制。建立村（社区）-街道（乡镇）-区（县）三级社会工作服务体系，按照“有场地、有设备、有人员、有服务功能、有工作流程、有规章制度”的标准，加快推进乡镇（街道）社工站建设。推动乡镇（街道）社工站在困难群众帮扶、老年人服务、困境儿童关爱保护、社会支持网络构建、社区参与能力提升、社会工作机构与志愿服务组织培育等方面发挥作用，成为基层治理与服务的重要力量。
　　提升社会工作服务机构能力。制定社会工作服务机构标准和规范。引导社会力量举办和发展社会工作服务机构。优先发展以老年人、残疾人、困境儿童、农村留守人员、流动人口、家庭暴力受害人等为重点服务对象的社会工作服务机构，推进精神慰藉、教育辅导、婚姻家庭、矫治帮教、戒毒等领域社会工作，培养专业人才，实施品牌化建设。完善城乡社区、社会组织、社会工作“三社联动”机制，发挥好社区志愿者、公益慈善资源协同作用，促进社会工作专业力量参与社会治理。
　　扩大专业社会工作人才队伍。支持民政服务机构与基层民政经办机构、社区和社会服务机构设置社会工作岗位，加强社会工作专业人才配备和使用。支持高等院校加强社会工作专业学位教育，强化优化专业建设和相关领域课程设置。鼓励一线社会工作者通过培训和考试提高专业能力，获得相应职业资格。建立分级培养机制和分类培训体系，提高社会工作专业人才总量和水平。实现“社会工作人才＋志愿者”联动服务模式常态化，形成社会工作人才引领志愿者、志愿者协助社会工作人才开展服务的良性互动机制。

　第五节　促进慈善事业健康发展

积极发挥慈善在第三次分配中的重要作用。配合全国人大常委会开展《[中华人民共和国慈善法](https://www.pkulaw.com/chl/5df107e39adb063fbdfb.html)》修改工作，出台关于发挥第三次分配作用进一步促进慈善事业健康发展的政策措施。加强社会救助、社会福利制度同慈善事业制度有机衔接，充分发挥慈善事业在保障改善民生中的积极作用。鼓励和支持慈善力量积极参与重大国家战略实施。健全慈善力量参与应对突发事件的体制机制。
　　鼓励慈善组织和慈善信托发展。推动《[中华人民共和国慈善法](https://www.pkulaw.com/chl/5df107e39adb063fbdfb.html)》激励扶持政策落地实施，协调有关部门完善税收等优惠政策，探索建立慈善行为记录和激励机制，鼓励发展慈善组织和慈善信托。健全慈善表彰制度，鼓励社会公众、民营企业积极参与社会公益和慈善事业。加强慈善组织培育，完善内部治理机制和行业自律，提高行业公信力。优化完善慈善组织登记认定、年度报告、信息公开、公开募捐资格管理等制度，加强慈善活动监督，规范慈善主体行为，保障慈善财产合法使用。畅通慈善领域投诉举报渠道，加强监管检查力度，及时查处违法违规行为。
　　规范发展互联网慈善。健全促进互联网慈善发展的政策措施和监管体系，加大互联网慈善支持引导力度，激励互联网慈善模式创新、业态创新、管理创新、技术创新，督促指导互联网募捐平台透明化、规范化发展。

　第六节　健全志愿服务体系

加强志愿服务体系和社会参与平台建设。推动完善志愿服务政策法规体系，推进志愿服务标准制定和宣贯，协同推进志愿服务激励褒扬、保险保障等制度建设，配合完善新时代文明实践志愿服务体系。培育发展志愿服务组织。强化志愿服务与慈善项目、社会工作协同。推广应用全国志愿服务信息系统，推动乡镇（街道）、城乡社区依托社区综合服务设施、社会工作站建设志愿服务站点，为群众参与和接受志愿服务提供便利条件。加强志愿服务记录和证明出具管理，持续推进志愿服务信息数据的归集和管理。推动养老服务、儿童福利、未成年人保护、婚姻登记等机构和城乡社区设置相对稳定的志愿服务岗位，在社会救助、养老助残、儿童福利和未成年人保护等领域组织开展专项志愿服务活动，培育一批志愿服务品牌项目。

　第七节　加强行政区划和行政区域界线管理

健全完善行政区划政策体系。进一步健全完善行政区划调整专家论证、风险评估、征求意见、实地调查等工作制度，细化工作规程，通过规范行政区划调整酝酿、申报审批、组织实施程序，提升行政区划设置的科学性、规范性、有效性。严格设立市辖区、县级市的政策标准，推动各地制定设立镇（街道）标准，规范引导城镇型政区行政区划调整，为推动形成优势互补的高质量发展区域经济布局创造条件。
　　因地制宜优化行政区划设置。以城市群为单元，审慎研究中心城市、节点城市、重要轴线和廊道沿线地区的行政区划调整。围绕更好发挥中心城市和城市群辐射带动作用，建设现代化都市圈，合理优化沿大都市圈发展主要轴线的关键节点城市行政区划设置。完善乡、镇、街道行政区划设置，合理优化基层政区体系结构。进一步加强沿边沿海地区行政区划管理。
　　审慎调整设区市市辖区规模结构。综合考虑城市功能定位、现有规模、综合承载能力以及所在区域整体发展态势等多方面因素，综合评估拟调整县（市）经济社会发展基础条件、与中心城区联系紧密程度及历史人文背景等，严格稳慎把握设区市下辖县（市）改区。以常住人口规模为主要依据，以优化市域空间结构、提高行政效能、完善功能布局为导向，支持中心城市市辖区优化重组。完善中心城市重要经济功能区行政区划设置。
　　稳妥增设中小城市。根据不同地区发展阶段和发展特点，有序推动具有重要战略意义、发展条件成熟的地区设市，充分发挥设市对优化城镇发展布局和完善行政区划格局的双重作用。推动重要战略地区设市补点，在城市群范围内、重要中心城市周边地区增设县级市，打造生态宜居、功能复合的“微中心”，承接中心城市功能疏解，缓解中心城市发展压力。以主体功能定位为导向，培育发展集聚人口、产业的中小城市，统筹推进新型城镇化和乡村振兴。支持民族地区、特殊类型地区中发展基础相对较好、符合条件的县设市，加快培育中小城市，带动周边地区发展。稳步有序推动符合条件的县和镇区常住人口20万以上的特大镇设市。通过增设中小城市完善现代化城镇体系，培育发展新的经济增长点，促进农业转移人口就地、就近城镇化。
　　依法推进行政区域界线管理。按照有利于巩固勘界成果、保持行政区域界线走向明确、维护边界地区社会稳定的原则，依法推进省级、县级行政区域界线联合检查；积极稳妥做好行政区域界线风险隐患排查防范工作，及时处置因行政区域界线认定不一致等引发的边界争议；加强行政区域界线界桩管理和维护，及时修复更换损毁、移位界桩。做好行政区划变更后的行政区域界线勘定工作。深入推进平安边界建设，创新界线管理模式，提升行政区域界线信息化、智慧化、精细化管理水平，切实维护边界地区和谐稳定。

专栏5 社会治理体系和治理能力建设工程

01 城乡社区服务设施建设

推动城市社区综合服务设施全覆盖，加强村级综合服务设施建设。积极推动中

西部地区特别是乡村振兴重点帮扶县优先在易地扶贫搬迁集中安置社区、生态

移民搬迁安置社区建设城乡社区综合服务设施，加快补齐短板。创新城乡社区

综合服务设施运行机制，提高设施利用率。

02 城乡社区服务提质增效

支持各地建立政府购买社区服务长效机制，探索实施社区服务（电子）券项目，

对老年人、困难群众家庭社区服务消费予以适当补贴，实现社区养老、托育、

家政等服务“一券通用”。推动物业服务企业为社区居民提供多样化生活服务。

实施“互联网＋社区”行动，开展智慧社区建设，扩大智能感知技术应用，建

设开发智慧社区信息系统和移动客户端，推动社区服务智能化，提高城乡社区

服务效能。

03 社会组织孵化基地建设

高水平建设社会组织孵化基地，加大专业技能培训、管理经验输出、经费场地

提供、人才队伍建设等支持力度，重点培育行业性、公益性、枢纽型社会组织

和提供养老、助残、儿童福利和未成年人保护等服务的社会组织。

04 行政区划变更效果评估

健全工作机制，研究制定行政区划变更评估工作规范，根据区划调整类型，合

理设定评估时段、期数和评估手段。探索建立评估指标体系，指引行政区划调

整方案研究论证工作。推动各省（自治区、直辖市）按照《[行政区划管理条例](https://www.pkulaw.com/chl/7846b03cf53df019bdfb.html)

>实施办法》对本级政府审批的区划调整进行评估，对本辖区内由国务院审批的

行政区划调整地方开展自评。

　第四章　全方位强化基本社会服务，推动民生福祉达到新水平

加强家庭建设，优化婚姻收养登记，加强未成年人保护和留守儿童、困境儿童关爱，推进地名、殡葬等公共服务，合理拓展服务内容，提高服务质量，提升基本社会服务专业化、精准化水平。

　第一节　提升婚姻收养管理服务水平

优化婚姻登记管理服务。完善婚姻管理服务标准，健全涉外婚姻登记管理制度。推动建立婚介服务机构监管制度，规范婚介行业事中事后监管。建立健全弄虚作假骗取婚姻登记的纠错机制，加大整治力度。加强婚姻登记机关规范化建设，打造优质服务窗口。探索将颁证仪式引入结婚登记流程、推动实现婚姻登记“跨省通办”。
　　强化婚姻家庭辅导教育。加强婚前指导、婚前保健、婚姻家庭关系调适和离婚辅导，减少婚姻家庭纠纷。通过公益创投、政策扶持、经费补贴、政府购买服务等方式，引导社会组织、基层群众性自治组织和专业人才，面向社区开展家庭教育、反家暴教育、恋爱婚姻家庭观念教育、生活减压和社会支持等预防性专业服务。鼓励有条件的婚姻登记机关设置社会工作岗位，开展社会工作和志愿服务。推进婚前辅导教育服务平台建设，实现婚姻家庭辅导室（婚姻家庭纠纷调解室）在县级以上婚姻登记机关覆盖率达到80%。
　　推进婚俗改革。建立整治天价彩礼、铺张浪费、低俗婚闹、随礼攀比等不正之风的长效机制，营造良好社会风气。增加优秀婚俗文化产品和服务供给，鼓励和推广传统婚礼、集体婚礼、纪念婚礼，加强婚姻登记场所文化建设，倡导健康文明、简约适度的婚俗文化。推动有条件的地方设置婚姻文化展示厅、婚俗文化博物馆或婚姻家庭文化教育基地。开展好家风好家教好家训进家庭、进社区、进村庄、进校园、进企业活动。
　　完善收养登记管理。统筹国内收养和涉外收养，健全完善儿童收养登记制度体系。推动建设全国统一的收养信息系统。完善收养评估标准，全面实施收养评估。研究制定支持家庭收养儿童福利机构内病残儿童的政策。规范收养登记档案管理，优化收养登记服务。探索收养工作部门协同机制，定期通报涉外收养登记信息，协商处置收养疑难问题。

　第二节　健全未成年人保护体系

健全完善未成年人保护法规制度。落实“家庭监护为主体、社会监护为补充、国家监护为兜底”的监护制度，形成家庭、社会、政府“三位一体”的未成年人保护合力。加强国家兜底监护体制机制建设，建立健全民政部门长期监护和临时监护措施。完善未成年人生活保障、助医助学等政策。建立“发现报告、应急处置、评估帮扶、监护干预”的未成年人保护联动响应机制。推动各地制修订未成年人保护法规，形成上下衔接的法规政策体系。
　　有效发挥未成年人保护工作协调机制作用。建立健全未成年人保护工作协调机制，协调督促有关部门落实职责，及时督办、处置侵害未成年人合法权益事件，提升未成年人保护热点案件处置群众满意率，统筹推进未成年人保护工作。积极动员村（居）委会、社会组织、社会工作者和志愿者开展有利于未成年人健康成长的社会活动和服务，引导社会各界关心关注、积极参与未成年人保护工作。推动形成党委领导、政府主导、民政部门牵头、相关部门各司其责、社会力量广泛参与的未成年人保护工作格局。
　　强化未成年人监护能力建设。按照“家庭尽责、政府主导、社会参与”的原则，强化家庭主体责任，加强家庭教育指导、监督、帮扶，提升家庭监护能力。完善应急处置机制，依托未成年人救助保护机构搭建民政和相关部门联动响应的一体化平台。建立监护评估机制，加强危机干预和家庭监护能力评估体系建设。指导村（居）委会等及时报告和处置监护人侵害未成年人合法权益事件。配合相关部门加大对不依法履行监护权的未成年人父母及其他监护人处置力度。

专栏6 未成年人保护能力建设工程

01 加强设施建设

加强未成年人保护设施建设。到2025年底，实现地级未成年人救助保护机构

全覆盖，县级未成年人救助保护机构覆盖率总体达到75%。提高未成年人救助

保护机构标准化、规范化水平。

02 提升服务能力

推动乡镇（街道）设立未成年人保护工作站或指定专门人员承担未成年人保护

工作，到2025年底，乡镇（街道）未成年人保护工作站或工作专干实现全覆

盖。推动村（居）委会设立专人专岗。加大村（居）儿童主任培训力度，参训

率达到100%。

　第三节　加强困境儿童保障和留守儿童、留守妇女关爱

加强困境儿童分类保障。精准确定困境儿童保障标准，分类实施保障政策。建立完备的困境儿童信息台账，实现一人一档案，实行动态管理。完善因突发事件影响造成监护缺失儿童救助保护制度措施。加强对困难家庭的重病、重残儿童生活保障和救助工作。动员引导企业、公益慈善等社会力量参与困境儿童保障工作，营造全社会关心关爱困境儿童的良好氛围。
　　深入开展农村留守儿童关爱保护。加强农村留守儿童动态信息管理和档案建设，为农村留守儿童精准化管理、个性化服务提供数据支持。健全农村留守儿童监护指导制度措施，督促指导农村留守儿童监护人依法履行监护职责。推动因外出务工等原因在一定期限内不能完全履行监护职责的父母或其他监护人依法确定被委托照护人，指导其签订书面照护协议。指导外出务工父母或其他监护人加强与留守子女亲情关爱和日常联络沟通，确保每周联系和交流不少于1次。指导村（居）委会有效履行强制报告、预防干预等职责。积极引导社会力量参与，打造一批具有社会影响力的农村留守儿童关爱服务慈善项目。
　　开展农村留守妇女关爱行动。加强精神关爱和家庭教育支持，提升农村留守妇女履行家庭监护主体责任的意识。有效发挥农村留守妇女主体作用，引导农村留守妇女积极参与农村留守儿童、留守老年人关爱服务活动。会同相关部门加强指导，提升农村留守妇女就业创业能力，积极为农村留守妇女创业发展搭建平台、提供服务，实现农村留守妇女就近就地就业。提高农村留守妇女法律知识和意识，配合有关部门畅通法律援助受理渠道，加大对侵害农村留守妇女合法权益违法犯罪行为的打击和惩治力度。

　第四节　推进地名管理和公共服务体系建设

健全地名法规制度和体制机制。做好修订《地名管理条例》的有关工作，制定完善配套规章制度。修订完善地名领域国家标准和行业标准，健全地名标准体系。加强地名命名更名管理，完善工作程序，建立健全评估论证、征求意见和备案公告等制度，增强地名管理的科学性，促进地名管理规范有序。积极宣传推广标准地名，加强对地名使用的监督管理，引导社会各界规范使用地名。充分调动各部门、社会各界参与地名管理服务积极性。加大地名管理专业人才培养力度，建立地名管理专家库。参与联合国地名标准化工作，推进我国地名国际标准化建设。
　　大力发展地名公共服务。推进城乡地名设标工作，加强地名标志设置和质量管理，建立健全地名标志巡检制度，探索应用电子信息新技术开发具有电子定位和物联网功能的新型地名标志，拓展服务形式和内容，提升地名管理服务效能，构建系统完备、标准规范的地名公共服务体系，为社会提供更加准确、便捷的地名导向和信息服务。
　　积极开展地名文化保护和利用。健全地名文化遗产保护名录制度，完善地名文化遗产价值评定体系，推动建立地名文化遗产保护名录，加强地名文化遗产保护，传承和弘扬优秀传统地名文化。创新地名文化平台建设，探索地名文化保护弘扬形式，积极发展地名文化产业，丰富地名文化产品和服务。
　　实施区划地名信息化标准规范。在现有国家标准、行业标准基础上，制定“互联网＋区划地名”相关数据编码、格式标准、共享规范等标准规范，推动标准规范的应用和实施。制定符合地方实际需求的区划地名信息化地方标准。健全区划地名信息更新维护长效机制，完善中国·国家地名信息库，推动构建统一规范、各具特色、协调推进、上下贯通的国家-省-市-县四级地名信息库体系。
　　加强区划地名信息大数据应用。推动区划地名与相关部门、社会主体实现数据共享，服务支持经济社会发展。应用电子地图、遥感影像、三维仿真等技术手段，拓展利用云平台、大数据、物联网等技术方式，通过民政数据共享交换平台，为社会提供高效、优质、便捷的信息服务。有效利用人工智能、区块链等新技术，推动区划地名管理与服务创新。

专栏7 地名服务工程

01 地名信息服务

加强中国·国家地名信息库等各级地名信息库建设，利用互联网、大数据、人

工智能等技术手段提升地名管理服务效能，推动地名服务与互联网地图服务、

智慧城市建设、文化遗产保护等融合发展，充分发挥地名信息基础性、服务性

作用。

02 标准地名推广

加强地名用字、拼写管理，积极推进外国地名、国际公有领域地名译写标准化。

规范全国各类型地名标志标识，推动广播电视、公文证件、出版物、地图等领

域规范使用标准地名，在全社会推广标准地名。

03 地名文化宣传弘扬

持续推进《中国地名大会》节目，指导各地开展多样化地名文化宣传活动，利

用普及面广、传播速度快、可视化强的方式宣传弘扬中华优秀地名文化。

　第五节　提高康复辅助器具助老助残能力

构建现代康复辅助器具产业格局。实施康复辅助器具产业国家综合创新试点，推动在京津冀、长三角、粤港澳大湾区等区域形成康复辅助器具优势产业集群，引导成渝双城、中部地区、东北地区等发展康复辅助器具特色产业。提高康复辅助器具制造和配置服务的数字化、信息化、智能化水平，开展行业企业服务型制造试点和示范推广。
　　创新驱动产业升级。围绕康复辅助器具领域重大基础理论、关键核心技术及重要产品的创新需求，搭建产学研联合攻关平台，促进高校、企业、科研院所共同解决产业创新发展的痛点难点。支持国家专业机构与地方政府通过合作共建，打造产业创新中心、产品服务信息平台、中试转化基地、成果交易和检验检测认证平台等区域产业公共创新平台。
　　推动健全支付保障体系。编制出台重度失能人员基本康复辅助器具推荐目录，支持有条件的地方将康复辅助器具配置纳入长期护理保险保障范围，鼓励重度失能人员等使用适宜有效的康复辅助器具。以恢复健康、回归社会为原则，推动扩展工伤保险中的康复辅助器具支付范围，鼓励有条件的地方将基本治疗性康复辅助器具逐步纳入基本医疗保险支付范围。鼓励商业保险公司将康复辅助器具配置纳入保险产品支付范围。推动制定基本型康复辅助器具补贴制度。鼓励有条件的地方为失能、重残等特殊困难群体配置康复辅助器具。
　　增强现代服务能力。深化人工智能、大数据、5G等技术在康复辅助器具配置服务中的应用，开展云端适配、个性化智造、智能共享租赁等服务。依托现有养老助残设施，支持多种方式建立康复辅助器具科普展示、配置服务专区。鼓励有条件的地方开展康复辅助器具社区租赁服务试点，促进康复辅助器具进家庭进社区进机构。
　　完善产品质量管理体系。开展康复辅助器具监督管理体系研究。加快重点产品、服务和管理标准的制修订，完善康复辅助器具标准体系。进一步提升国家级康复辅助器具检验机构质量测评、仲裁检验等技术水平，支持建设质量检验区域示范中心和质量认证机构，鼓励在政府采购中充分发挥各级质检机构和认证机构的质量监督保障作用。

专栏8 康复辅助器具产业发展工程

01 提升区域产业创新能力

推动京津冀、长三角、粤港澳大湾区、成渝双城经济圈等区域设立4-6家康复

辅助器具区域科技创新基地和检测技术平台。鼓励其他区域统筹各类资源，布

局建设康复辅助器具科技创新中心和产业公共服务平台。

02 推广康复辅助器具示范应用

发挥国家级康复辅助器具机构资源优势，选取适合的养老助残机构，推动建设

高水平、广覆盖的康复辅助器具应用示范模式，开展康复辅助器具在多场景应

用、医养康养相结合、养老应急救援等方面的示范推广。开展康复辅助器具公

益宣传活动，提高功能障碍群体自主自助选择和使用康复辅助器具的能力。

03 发布新版《中国康复辅助器具目录》

修订《中国康复辅助器具目录（2014版）》。编制《中国康复辅助器具推广目

录》。

04 建设产业公共服务平台

建设康复辅助器具产业公共服务平台，提高全产业信息化服务能力，加强养老

助残行业信息沟通。

　第六节　完善殡葬公共服务体系

全面建立基本殡葬服务保障制度。切实履行政府对城乡困难群众基本殡葬服务兜底保障职责，以减免或补贴方式提供遗体接运、暂存、火化、骨灰存放等基本殡葬服务。提高政府基本殡葬服务供给能力，有条件的地方可增加服务项目、扩大覆盖人群和提高保障标准，鼓励优先将生态安葬纳入本地基本公共服务范围。
　　加快补齐殡葬服务设施短板。立足殡葬改革目标，统筹群众需求和设施短板，科学确定火葬区殡葬服务设施设备建设或配置标准，支持新建殡仪馆或公益性骨灰安放设施，尚未覆盖的县市补齐短板，有条件的地方立足实际加强现有殡葬设施设备改造提升。合理规划建设土葬改革区遗体公墓，统筹设置服务土葬的殡仪服务设施（场所），推动形成覆盖城乡、设施完善、供给充足、火葬土葬改革一体推进的殡葬服务网络。制定公墓建设管理办法和节地生态安葬标准，推动公益性墓地建设，鼓励和引导绿色环保用材、节约用地、生态安葬。
　　切实提高殡葬领域治理水平。根据人口、耕地、交通情况，实事求是地划分火葬区和土葬改革区，并适时进行调整优化。积极稳妥推进活人墓、豪华墓、乱埋乱葬治理，遏制增量、减少存量。规范和加强殡葬服务管理，落实属地管理责任和部门监管责任，强化殡葬服务事业单位公益属性，规范殡葬中介机构、服务企业经营行为，建立健全综合监管机制。推动殡葬服务与“互联网＋”融合，探索推广远程告别、网络祭扫等殡葬服务新模式，提升殡葬服务便捷化、智能化水平。健全殡葬领域人才培养和激励机制。深入推进丧葬习俗改革，充分发挥党员干部模范带头作用，健全红白理事会及村规民约，传承发展优秀殡葬文化，遏制重殓厚葬等陈规陋习。

专栏9 殡葬服务设施建设工程

01 加强殡仪馆建设

支持火葬区殡仪馆尚未覆盖的县市补齐短板。压实地方责任，对已达危房标准、设施设备陈旧的殡仪馆实施改扩建，对已达到强制报废年限或不符合国家环保标准的火化设备进行更新改造，推动基本殡葬服务设施覆盖全部县市。

02 推动公益性骨灰安放设施建设

支持公益性骨灰安放设施建设。推进农村公墓建设。加大生态殡葬奖补力度，

在部分地区实施节地生态型公益性公墓示范项目。依托现有设施或适当场所，为不保留骨灰者和遗体器官捐献者建设纪念载体。

　第五章　全要素构建养老服务体系，在实施积极应对人口老龄化国家战略中彰显新作为

适应人口老龄化社会需求，持续完善居家社区机构相协调、医养康养相结合的养老服务体系，健全失能老年人长期照护服务体系，强化信用为基础、质量为保障、放权与监管并重的服务管理体系，优化供给结构、提升服务质量，有效强化基本养老服务，满足多样化、多层次养老服务需求。

　第一节　加强养老服务保障

加强基本养老服务。建立基本养老服务清单，逐步丰富发展服务项目。制定并实施全国统一的老年人能力综合评估标准，开展老年人能力综合评估，评估结果作为领取老年人补贴、接受基本养老服务的参考依据。完善经济困难的高龄、失能老年人补贴制度，因地制宜健全动态调整机制，提高补贴标准精准度和有效性，强化与残疾人“两项补贴”、社会救助等政策的衔接。
　　完善兜底性养老服务。做好城乡特困人员集中供养工作，提升特困人员供养服务设施（敬老院）集中供养和失能照护能力。以重残、失能、留守、空巢、计划生育特殊家庭等特殊困难老年人为重点，建立特殊困难老年人定期探访制度、农村留守老年人关爱服务制度。
　　发展长期照护保障。推动建立长期照护服务项目、标准、质量评价等行业规范和全国统一的老年人能力评估体系。配合有关部门稳妥推进长期护理保险制度试点，推动完善长期护理保险制度，逐步扩大长期护理保险参保群体范围。充分发挥社会救助、社会福利、慈善事业、商业保险有益补充作用，保障不同层面照护需求。

　第二节　优化居家社区机构养老服务网络

完善居家养老支持措施。推动各地建立家庭养老支持政策，支持具备条件的养老服务机构运营社区养老服务设施。健全建设、运营、管理政策，发展“家庭养老床位”。推动失能失智和高龄老年人家庭成员照护培训纳入政府购买养老服务目录，推进经济困难高龄老年人家庭适老化改造。探索“物业服务＋养老服务”模式，支持有条件的地区探索开展失能失智老年人家庭照护者喘息服务。
　　优化社区养老服务设施布局。大力推进城市新建城区、新建居住（小）区按标准配套建设社区养老服务设施，配建设施达标率达到100%。推进老旧城区、已建成居住（小）区基本补齐社区养老服务设施，推动构建城市地区“一刻钟”居家养老服务圈，努力解决大城市养老难问题。加强社区养老服务设施用途管理，支持将社区养老服务设施低偿或无偿用于普惠型养老服务。
　　实施老年人居家和社区适老化改造工程。实施特殊困难老年人家庭适老化改造，引导有需求的老年人家庭开展适老化改造，配合做好老旧小区适老化改造工作。支持社区养老设施配备康复辅助器具并提供专业指导。鼓励基层群众性自治组织、社区养老服务机构、老年人协会、企事业单位开展社区互助养老活动。
　　推进区域养老服务中心建设。在县（市、区、旗）、乡镇（街道）范围推动区域养老服务中心建设，发展具备全日托养、日间照料、上门服务、区域协调指导等综合功能的区域养老服务机构。根据人口分布、老龄化水平及发展趋势布局建设层次清晰、功能互补、区域联动的养老服务网络。
　　推动养老机构提质发展。深化公办养老机构改革，发挥公办养老机构兜底线、保基本和示范作用。引导社会办养老服务机构重点面向中低收入群体、适度面向中高收入群体，满足老年人多层次、个性化服务需求。优化城乡养老机构床位结构，推动护理型床位占比不低于55%。引导养老机构依托新兴技术手段，构建“互联网＋养老服务”和智慧养老模式，培育服务新业态。鼓励养老机构向家庭提供生活照护、康复护理、精神慰藉、紧急救援等专业服务。鼓励有条件的公办养老服务设施通过承包经营、委托运营、联合经营等方式，引入企业或社会服务机构参与运营管理。
　　积极推进医养结合服务。推动社区养老服务设施与社区卫生服务站、护理站或其他医疗设施邻近设置。有条件的养老机构可按相关规定申请开办康复医院、护理院、中医医院、安宁疗护等机构。健全养老机构与医疗卫生机构合作机制，推动医疗卫生机构为养老机构开通就诊绿色通道。
　　提升应急救援能力。构建“分层分类、平战结合、高效协作”的养老服务应急救援体系。建立国家养老应急救援技术服务中心，加强对地方养老应急救援力量的技术支持和服务。推动建立区域性养老应急救援技术服务中心，负责为本区域养老服务应急救援体系建设提供服务。
　　大力发展农村养老服务。构建乡镇牵头，村委会、老年人协会、低龄健康老年人、农村留守妇女、村干部、党员、志愿者等广泛参与的农村互助养老服务格局。完善农村留守老年人关爱服务体系。大力培育农村为老服务社会组织，强化农村老年人社会支持体系建设。鼓励农村集体建设用地优先用于发展养老服务，逐步提高农村养老服务设施的建设补贴、运营补贴水平。支持民族地区、特殊类型地区因地制宜发展农村养老服务。
　　推进养老服务适度普惠。大力发展普惠养老服务，为中低收入家庭老年人提供价格适中、方便可及、质量可靠的养老服务。吸引更多社会力量参与开展普惠养老服务，推动中央、省、市各级党政机关和国有企事业单位培训疗养机构转型为养老服务设施，并支持其可持续发展。支持国有经济加大对养老服务的投入，积极培育发展以普惠型养老服务等为主责主业的公共服务功能国有企业。

　第三节　壮大养老服务产业

做强养老产业主体。发展银发经济，实施“养老服务＋行业”行动，促进文化、旅游、餐饮、体育、家政、教育、养生、健康、金融等行业与养老服务融合发展，培育智慧养老等新业态，促进养老企业连锁化、集团化发展。大力培育养老服务行业组织，发挥行业协会积极作用。
　　促进老年用品开发。加快完善老年用品标准，推动老年用品生产研发，支持新兴材料、人工智能、虚拟现实等新技术在养老服务领域的集成应用与推广。开发适老化技术和产品，重点发展适老康复辅助器具、智能穿戴设备、服务型机器人与无障碍科技产品。
　　培育老年消费市场。健全行业标准和市场规范，营造平等参与、公平竞争的市场环境；加大市场监管力度，营造安全、便利、诚信的消费环境。推动老年用品进家庭、社区、机构和园区，带动产品供给和产业发展。逐步推动社会力量成为发展养老服务业的主体，促进老年消费市场的繁荣与发展。

　第四节　加强养老服务人才队伍建设

健全人才教育培训体系。推进养老服务相关专业教育体系建设，鼓励养老机构为有关院校教师实践和学生实习提供岗位，参与专业建设，提高产教融合水平。支持养老服务相关专业开展职业教育校企合作，鼓励符合条件的养老机构举办养老服务类职业院校。持续实施养老服务人才培训提升行动，大规模培训养老护理员、养老院院长、老年社会工作者。推动实现每千名老年人、每百张养老机构床位均拥有1名社会工作者。
　　发展养老护理员队伍。开展养老护理员职业技能提升行动，到2022年底，培养培训200万名养老护理员。推动建立健全养老护理员职业技能等级认定制度。建设全国养老护理员信息和信用管理系统，支持有条件的地区制定入职补贴、积分落户、免费培训、定向培养和工龄补贴等优惠政策。举办养老护理员职业技能竞赛，开展养老护理员表彰活动。

　第五节　提升综合监管水平

健全综合监管制度。按照“谁审批、谁监管；谁主管、谁监管”原则，建立职责明确、分工协作、科学有效的综合监管制度，建立跨部门综合监管体制。实施全国养老机构服务质量等级评定，提升养老机构规范化水平。建立“养老服务＋信用”机制，建立覆盖从业人员和服务对象的信用管理体系。
　　推进标准化建设。制定一批与国际接轨、体现本土特色、适应管理服务要求的养老服务行业标准。推动地方标准、团体标准上升为国家标准、行业标准，鼓励养老机构制订高于国家标准的企业标准。加快建立全国统一的养老服务质量标准和评价体系。推动养老机构和行业组织实施标准化管理。
　　加强风险监测与防控。建立健全养老服务安全风险分级管控和隐患排查治理的制度规范，实现养老服务机构安全风险自辨自控、隐患自查自治，提升安全生产整体防控能力。落实养老服务领域非法集资处置工作行业部门责任，建立协同机制。发挥“金民工程”全国养老服务信息系统作用，建立养老服务机构信息联动机制，加大风险排查力度。开展常态化非法集资识骗防骗教育宣传，增强老年人群体识别防范能力。

专栏10 养老服务设施建设工程

01 特殊困难家庭适老化改造

聚焦特殊困难高龄、失能、残疾老年人需求，以施工改造、设施配备、老年用

品配置等形式，支持200万户困难家庭实施适老化改造。

02 居家社区养老服务网络建设

支持一批区县建设连锁化运营、标准化管理的示范性居家社区养老服务网络，

形成街道层面区域养老服务中心和社区层面嵌入式养老服务机构衔接有序、功

能互补的发展格局，为居家社区老年人提供失能护理、短期托养、心理慰藉等

服务。

03 区域养老服务中心建设

支持有条件的特困人员供养服务设施（敬老院）转型为区域养老服务中心，推

动在乡镇（街道）范围内建有具备全托、日托、上门服务等综合功能的养老服

务设施，到2025年底，区域养老服务中心在乡镇（街道）的覆盖率总体达到60%。

04 养老机构护理能力提升

支持县（市、区、旗）建设提供专业化失能照护服务的养老服务机构。支持1000

个左右公办养老机构增加护理型床位。支持300个左右培训疗养机构转型为普

惠养老机构。

05 应急救援能力提升

新建和改扩建若干具备综合应急救援能力的养老服务设施，配备必要的防疫物

资和设施设备。推动具备条件的公办养老机构配备应急防护物资、隔离设施，

建立完善应急预案，培养培训专业养老服务应急队伍，定期分类开展应急演练，

加强应急能力建设。鼓励有条件的民办养老机构提升应急救援能力。

　第六章　强化实施保障，推进民政事业高质量发展

坚持和加强党的全面领导，推进法规、制度、标准体系建设，加强资金保障，加强人才队伍、基层能力建设和新技术应用，构建全方位、多层级、管长远的民政事业发展保障体系，确保规划顺利实施，目标任务如期完成。

　第一节　全面推进民政系统党的建设

各级民政部门要深入学习贯彻习近平新时代中国特色社会主义思想，增强“四个意识”、坚定“四个自信”、做到“两个维护”，切实践行“民政为民、民政爱民”工作理念，积极创建“让党中央放心、让人民群众满意”的模范机关，把党的领导贯彻规划实施各领域、全过程。不断提高政治判断力、政治领悟力、政治执行力，积极探索党建工作和业务工作同向而行、同频共振的方法路径，确保党建工作和业务工作深度融合、互促共进。健全民政系统责任落实机制，聚焦巩固拓展脱贫攻坚兜底保障成果同乡村振兴有效衔接，聚焦特殊群体，聚焦群众关切，推进民政工作不断开创新局面。加强干部队伍建设，提高民政系统领导班子和各级干部适应新时代新要求，抓改革、促发展、保稳定的能力和水平。坚定不移推进全面从严治党，坚持无禁区、全覆盖、零容忍，一体推进不敢腐、不能腐、不想腐，不断涵养民政系统风清气正的良好政治生态，坚决整治侵害群众利益的腐败问题，强化政治监督，为实现规划目标任务，推动民政事业高质量发展提供坚强政治保障。

　第二节　加强民政法治化标准化建设

健全民政法治体系。深入学习宣传贯彻习近平法治思想，全面贯彻落实中央关于法治中国、法治社会、法治政府决策部署，全面推进依法行政，为民政事业蓬勃发展提供坚强法治保障。深入实施《[中华人民共和国民法典](https://www.pkulaw.com/chl/aa00daaeb5a4fe4ebdfb.html)》、《[中华人民共和国慈善法](https://www.pkulaw.com/chl/5df107e39adb063fbdfb.html)》、《[中华人民共和国村民委员会组织法](https://www.pkulaw.com/chl/09d5ef876a515fe8bdfb.html)》、《[中华人民共和国城市居民委员会组织法](https://www.pkulaw.com/chl/74b469aedc9b119fbdfb.html)》、《[中华人民共和国残疾人保障法](https://www.pkulaw.com/chl/bae1a775ddfaf017bdfb.html)》、《[中华人民共和国老年人权益保障法](https://www.pkulaw.com/chl/60ff5933d32dc7ccbdfb.html)》、《[中华人民共和国未成年人保护法](https://www.pkulaw.com/chl/883cecd7fe38636dbdfb.html)》、《[志愿服务条例](https://www.pkulaw.com/chl/49a4eb2d1000a625bdfb.html)》、《[行政区划管理条例](https://www.pkulaw.com/chl/7846b03cf53df019bdfb.html)》等法律法规，开展法律法规有关规定落实情况跟踪评估。积极推进社会救助、社会事务、社会组织、基层群众性自治组织等重点领域法律法规的制定和修订工作。贯彻落实《[重大行政决策程序暂行条例](https://www.pkulaw.com/chl/57ea6b1fe43a223cbdfb.html)》，充分发挥法律顾问、公职律师在重大行政决策中的作用。强化民政行政执法能力建设，完善行政执法各项制度，改进和创新行政执法方式，加强行政执法监督，扎实做好行政复议和应诉工作，加大对违法和不当行政行为的纠错力度。进一步完善学法用法普法制度，强化法治培训，不断增强各级领导干部依法办事的意识和能力。
　　建立完善民政标准体系。坚持深化改革与创新驱动相结合、整体推进与重点突破相结合、制定修订与宣传实施相结合、标准化建设与法治化建设相结合，深度推进标准化与业务工作相融合，研究制定工作标准和制度流程并组织推广，为民政事业现代化建设提供技术支撑和服务保障。健全通用基础标准、业务专用标准、技术支撑标准，形成科学合理、层次分明、配套全面、功能完备的民政标准体系。加大培训力度，推广民政标准应用。依托现有资源，建立民政标准信息平台，实现国家标准、行业标准、地方标准等信息互通共享。加大养老助残等领域先进国际标准转化力度，开展民政标准化基础理论和通用方法研究，推动研究成果转化为国家和行业标准。

　第三节　提升民政信息化水平

推动提升政务服务效能。坚持一盘棋布局、一张网建设、一体化发展，加快推进电子政务建设，通过流程优化、功能重组、数据集成等方式，健全完善各类民政政务信息系统，推动建设“金民工程”二期项目。深化“互联网＋民政服务”，提高民政政务服务平台供给能力，拓展空间位置服务和便民惠民地图服务，推动更多民政政务服务事项“一网通办”、“跨省通办”、“就近可办”，实现网上可办率达到80%。保留对老年人等特殊群体的线下窗口和服务渠道。充分利用12345热线资源体系，进一步畅通同群众、企业的互动渠道，提升民政政务服务便捷度，增强群众获得感。
　　充分发挥数据要素作用。加强数据资源全要素、全口径、全生命周期管理，促进数据、政策、技术、服务等相互融通、上下一体，全面构建以行政区划代码、社会组织和村（居）委会统一社会信用代码等为基础，以自然人、法人为核心的民政大数据资源体系。完善数据标准，有效拓展数据采集广度和深度，推进数据在电子政务内网外网普遍共享、综合应用。
　　优化完善应用支撑平台。深度集成融合大数据、人工智能、区块链、GIS等技术，升级优化智慧民政全业务应用支撑平台。打造功能重组、服务重用、组织重构的基础应用支撑平台，构建集数据治理、资产管理、数据建模、数据服务等于一体的大数据平台，建设集语音识别、语义识别、人脸识别、画像分析等于一体的人工智能平台，提升业务拓展能力、大数据分析能力、决策支持能力。
　　加快信息基础设施建设。构建多网融合、稳定运行、安全可信的民政网络和信息基础设施大平台，提供统一云服务和安全保障。全面提升由民政政务云中心、大数据中心、异地灾备中心构成的“两地三中心”信息基础设施的网力、算力和承载力，加大安全可靠技术产品应用，加快民政信息基础设施高可靠、高性能、高安全、智能化升级，建设网络安全统一运维管控与综合防范业务平台。

　第四节　优化民政资源配置

加强资金保障。加强兜底性、基础性民政工作经费保障，与经济社会发展水平相适应，与宏观政策、社会政策相衔接。完善转移支付分配管理，向中西部地区、脱贫地区、民族地区、特殊类型地区等倾斜。优化中央财政安排用于社会福利事业的彩票公益金分配使用，提高资金使用效益。扩展民政事业资金来源渠道，总结金融支持养老服务的有益经验，综合运用税费减免、资金引导、培育孵化、人才支持、精神奖励、共建共治等多种手段，广泛吸引各类社会资金投入。加强民政服务设施建设，引导建设资金投向短板领域和薄弱环节。在保障实际需求的基础上，加强养老服务、儿童福利、未成年人保护、救助管理、精神卫生福利等服务设施资源整合和统筹利用，节约成本提高效率。
　　推动对外合作交流。依托“一带一路”等多边、双边机制，加强社会救助、社区治理、地名管理、养老服务、儿童福利、社会事务、慈善事业、社会工作、志愿服务、康复辅助器具等领域的对接合作，讲好中国故事，实现互利共赢。拓展与港澳台地区交流合作；畅通沟通渠道，完善海峡两岸婚姻家庭服务。引导规范社会组织依法有序参与国际交流合作。
　　强化干部队伍建设。着力建设政治过硬、具备领导民政现代化建设能力的民政骨干队伍。坚持以党的政治建设为统领加强领导班子建设，着力提高干部队伍专业化能力，严格干部管理监督，以正确的选人用人导向激发干事创业的热情和责任担当，强化对敢担当善作为干部的激励和保护。持续抓好干部培养工作，在困难艰苦地区和基层一线锻炼干部。完善继续教育体系，健全专业人才职称评定制度，培育造就一支专业化、职业化民政技术技能人才队伍。
　　增强基层服务能力。推动民政服务事项清单化管理，规范完善服务内容和标准，强化培训指导，提升基层民政为民办实事能力。总结推广乡镇（街道）社会工作人才队伍建设经验，推动乡镇（街道）社工站全覆盖，打通为民服务“最后一米”。推动健全政府购买社会工作服务长效机制，促进乡镇（街道）社工站建设和增强基层民政服务能力融合发展。

　第五节　统筹民政领域发展和安全

切实强化安全意识。坚持总体国家安全观，牢固树立安全发展理念，坚持以人民为中心，切实强化底线思维，构建民政大安全格局。坚决捍卫政治安全，维护国家安全。深入贯彻落实党中央、国务院关于安全生产工作决策部署，把安全发展理念贯穿民政事业各领域和全过程，坚守“三个必须”和“谁主管谁负责”的原则，建立健全民政领域风险防范化解工作机制，严格落实安全生产责任制，完善安全预防控制体系，健全安全风险评估制度，加强安全生产宣传教育培训，持续开展安全隐患排查整治。健全安全生产应急处置工作机制，提升应急救援能力，健全完善预案体系，定期组织演练，确保发挥作用。
　　全面做好疫情防控。深入学习贯彻习近平总书记关于统筹疫情防控和经济社会发展重要论述精神，坚持常态化精准防控和局部应急处置有机结合，不断巩固民政领域疫情防控成果。督促指导民政服务机构完善常态化监测预警工作措施，加强机构内老年人、儿童、特困人员等健康监测。不断改善民政服务机构、城乡社区防控设施和条件，提升应急救援能力。建立跨区域援助工作机制，根据需要对疫情防控压力大、能力相对薄弱的地区及时开展支援。

　第六节　完善规划实施监测评估机制

强化主要目标、重点任务、重大工程的分解落实，完善配套政策。建立年度监测分析、中期评估和总结评估的规划实施监测评估工作机制，将评估结果作为改进民政工作和绩效考核的重要依据。健全统计指标体系，强化对重点领域、重点区域的动态监测。创新评估方式方法，通过引入第三方机构、综合运用大数据分析等方式，丰富评估维度，提升评估深度。畅通群众诉求和意见表达渠道，发挥群众监督、媒体监督的积极作用。各级民政部门要强化主体责任，以本规划为指导，因地制宜制定本地区民政事业发展规划或行动计划，对本规划和本地区民政规划计划的落实情况开展监测评估，对实施过程中出现的问题要及时上报。各级发展改革部门要立足职责职能，通过发展规划、年度计划、投资引导等政策手段，支持规划目标任务落地见效。确需对本规划的重大目标任务进行调整时，由民政部协商国家发展改革委制定调整方案。

© 北大法宝：（[www.pkulaw.com](https://www.pkulaw.com/)) 专业提供法律信息、法学知识和法律软件领域各类解决方案。北大法宝为您提供丰富的参考资料，正式引用法规条文时请与标准文本核对。
欢迎查看所有[产品和服务](https://www.pkulaw.com/helps/2.html)。法宝快讯：[如何快速找到您需要的检索结果？](https://www.pkulaw.com/helps/70.html)[法宝](https://www.pkulaw.com/helps/69.html)V6有何新特色？

扫描二维码阅读原文

**原文链接：**<https://www.pkulaw.com/chl/b6e430ad8258d8e3bdfb.html>
